# Mechanism-guided mitigation of acrylamide in thermally processed foods: emerging technologies and analytical verification

**DOI:** 10.3389/fnut.2026.1981497

**Published:** 2026-10-20

**Authors:** Tianrui Liu, Kai Zhang, Qingqing Yuan, Chenhui Ren, Moyan Ji, Kailin Lin, Chenguang Zhou, Xiaobo Zou

**Affiliations:** School of Food Science and Engineering, Jiangsu University, Zhenjiang, China

**Keywords:** acrylamide, analytical verification, food matrix, mechanism-guided mitigation, thermal processing

## Abstract

Acrylamide (AA) forms mainly from free asparagine and reducing sugars during high-temperature food processing. Experimental evidence of AA neurotoxicity and glycidamide-mediated genotoxicity supports mitigation, although the association between dietary AA exposure and human cancer remains inconclusive. This narrative review evaluates how formation mechanisms and matrix-specific constraints should guide AA mitigation and how reported reductions should be verified. The evidence indicates that effectiveness is constrained by precursor accessibility and mass transfer, enzyme–substrate contact, and the thermal–moisture history of the complete process. Pulsed electric fields and ultrasound may mobilize precursors, but lowering their availability requires effective aqueous removal; high-pressure processing may improve enzyme access, while vacuum and alternative heating alter thermal exposure and moisture removal. Direct AA evidence for cold atmospheric plasma remains specific to tested food and discharge conditions, whereas plasma-activated water is not yet established as an AA-control operation. Reported percentage reductions cannot rank technologies across different matrices, processing sequences, and final-product states; likewise, combined-treatment effects do not establish synergy without matched comparisons of individual and combined operations. Liquid chromatography–tandem mass spectrometry provides confirmatory AA quantification, whereas rapid assays and spectroscopic techniques are better suited to screening and process monitoring. This review therefore frames AA control as a matrix-specific process-design problem in which operations with distinct mechanistic targets are selected and their incremental effects are assessed through direct AA quantification under matched final-product conditions for moisture, color, and texture.

## 1 Introduction

The safety of agricultural products is influenced not only by constituents already present in raw materials at harvest, but also by contaminants formed during processing. Acrylamide (AA) is generated during high-temperature food processing (1, 2); in carbohydrate-rich food systems, its principal formation pathway is the Maillard reaction between free asparagine and reducing sugars (3, 4). Potato products, cereal-based foods, and coffee are important sources of dietary AA exposure (5). AA concentrations in finished products depend on precursor levels and their changes during storage and are further influenced by formulation, food-matrix structure, moisture migration, and processing thermal history (6). AA control therefore involves both raw-material management and process design.

Experimental evidence supports AA neurotoxicity and the genotoxicity mediated by its metabolite glycidamide, providing a basis for AA mitigation; however, evidence from human studies on the association between dietary AA exposure and cancer remains inconclusive (5). Hazards observed under experimental conditions should not be directly equated with health effects at typical dietary exposure levels. Mitigation measures must also account for food quality, because thermal reactions associated with AA formation contribute to the color, aroma, and texture of baked, fried, and roasted foods (7). Free asparagine and reducing sugars are the principal determinants of AA formation potential, whereas pH, water activity, food structure, and local heating conditions influence the extent to which this potential translates into actual AA formation (7, 8). Accordingly, even under nominally similar heating conditions, final AA concentrations may differ when precursor levels or moisture-change trajectories differ (9).

Existing mitigation measures include raw-material selection and storage management, washing or blanching, fermentation, asparaginase treatment, acidification, and adjustment of heating conditions (7, 8). Research on pulsed electric field, ultrasound, cold atmospheric plasma, vacuum processing, and microwave heating has expanded the available options for controlling AA by altering precursor accessibility, surface chemistry, or heat and moisture transfer (10–14). However, the percentage reductions reported in the literature reflect outcomes obtained under specific food-matrix, treatment-sequence, and final-product conditions and should not be regarded as the intrinsic mitigation efficacy of a technology. The evidence from studies that directly measure AA also varies across methods. The central question is therefore which dominant limiting factor a given intervention can alleviate in a target product: precursor availability, mass transfer, enzyme accessibility, or exposure to temperature and moisture conditions conducive to AA formation (15, 16).

Reliable analytical validation is also needed to determine whether a selected intervention actually reduces AA. The physicochemical properties of AA and interference from food-matrix components make its quantification sensitive to sample preparation and analytical selectivity. Chromatography–mass spectrometry methods can be used for confirmatory AA determination, whereas rapid methods and optical techniques can support screening and process monitoring, provided that their performance has been evaluated for the relevant food matrix (17–20). Interpretation of observed AA reductions should also consider the complete treatment sequence, appropriate controls, and comparable final-product endpoints.

This narrative review aims to evaluate how AA formation mechanisms and matrix-specific limiting factors can guide the selection of mitigation measures and how analytical measurements can validate the resulting mitigation conclusions. It links the toxicological basis and principal formation pathways to intervention targets, distinguishes the roles of confirmatory quantitative analysis from those of screening and process-monitoring tools, and evaluates processing strategies in terms of their mechanisms of action, evidence boundaries, and applicability to different food matrices and production lines. For combined treatments, the review gives particular attention to the required controls and matched final-product endpoints needed to avoid inferring synergistic effects from an overall AA reduction alone.

## 2 Health relevance of acrylamide as a basis for mitigation

### 2.1 Absorption, metabolism, and glycidamide formation

Following oral ingestion, AA is absorbed through the gastrointestinal tract and distributed to tissues throughout the body (Figure 1A). Its metabolism proceeds mainly via two pathways: conjugation with glutathione (GSH), followed by conversion to mercapturic acid metabolites that are excreted in urine, or oxidation to glycidamide (GA) (5). Mouse studies indicate that cytochrome P450 2E1 (CYP2E1) is the principal enzyme catalyzing the conversion of AA to GA, which can form adducts with deoxyribonucleic acid (DNA) and hemoglobin (21). Figure 1B compares the conjugation-mediated detoxification pathway that promotes *in vivo* elimination with the bioactivation pathway associated with GA-related genotoxicity.

**Figure 1:**
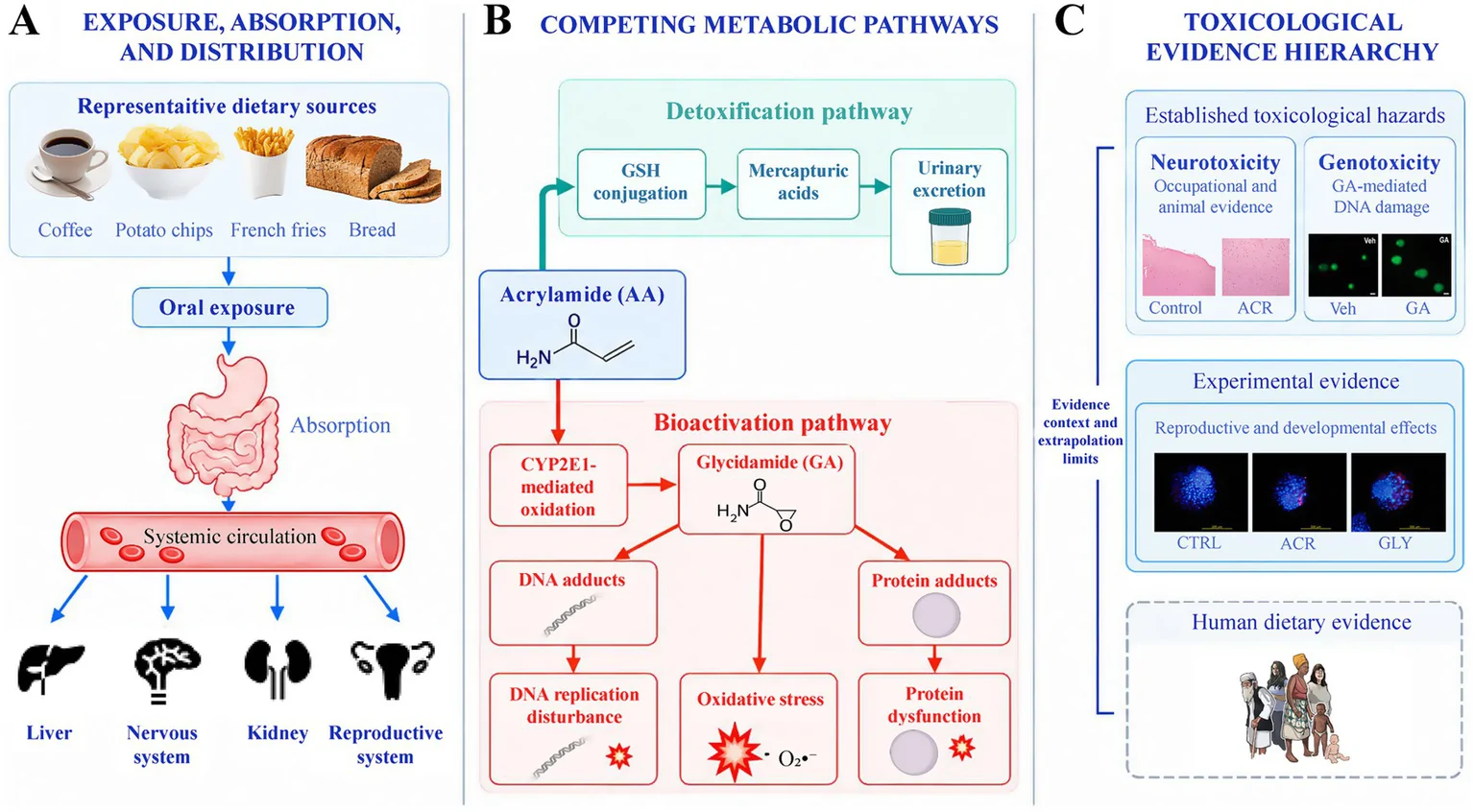
Dietary exposure, absorption, and distribution of acrylamide **(A)**; competing metabolic pathways **(B)**; and the toxicological evidence hierarchy **(C)**. In panel C, representative cortical histology images illustrating acrylamide-associated neurotoxicity were adapted from Figures 11A,B of Alshammari et al. (203); Veh and GA comet-assay images illustrating GA-mediated DNA damage were adapted from Figure 4A of Walton et al. (204); and representative merged DAPI/TUNEL blastocyst images illustrating reproductive and developmental effects were adapted from Figure 7C of El-Sheikh et al. (26). All images were adapted under the Creative Commons Attribution License (CC BY 4.0), with cropping and layout adjustments for consistency. AA/ACR, acrylamide; GA/GLY, glycidamide; Veh, vehicle; CYP2E1, cytochrome P450 2E1; GSH, glutathione. The four organ icons were obtained from [Health Icons](https://healthicons.org/about) and are licensed under CC0. The [DNA](https://bioart.niaid.nih.gov/bioart/125), [protein](https://bioart.niaid.nih.gov/bioart/481), and [human population](https://bioart.niaid.nih.gov/bioart/185) illustrations in Figure were obtained from NIAID NIH BioArt Source.

### 2.2 Major toxicological effects

Neurotoxicity was among the earliest toxic effects of AA to be identified (22). Observations from occupational exposure and animal studies have linked AA exposure to ataxia, muscle weakness, sensory impairment, and peripheral nerve damage. These neurotoxic manifestations may involve impaired axonal and synaptic function, as well as oxidative stress; however, uncertainty remains as to whether the mechanistic evidence can be extrapolated to chronic exposure at typical dietary levels (23).

GA-mediated DNA damage provides a mechanistic basis for AA genotoxicity. Experimental studies indicate that a specific derivative of a GA-DNA adduct can impede DNA replication and induce mutations (24). Following its 2022 re-evaluation, the European Food Safety Authority (EFSA) concluded that new evidence supports its previous conclusions on AA genotoxicity and that the margin of exposure approach remains appropriate for characterizing the risk of neoplastic effects (25).

Beyond neurotoxicity and genotoxicity, the reproductive and developmental toxicity of AA has also attracted attention, although the relevant evidence is derived primarily from experimental models. For example, exposure of bovine oocytes to AA or GA during *in vitro* maturation reduced their subsequent developmental competence (26). However, this finding is limited by the experimental model and exposure conditions and cannot be directly extrapolated to human dietary exposure scenarios.

The primary sources of evidence for these toxicological endpoints and their limitations for extrapolation are summarized in Figure 1C, including observations from occupational exposure, animal studies, and mechanistic and cellular studies. This evidence provides a basis for identifying potential AA hazards but is insufficient for directly comparing the magnitude of risks associated with different health effects under typical dietary exposure levels.

### 2.3 Dietary exposure and risk interpretation

Dietary exposure to AA depends on AA concentrations in foods and dietary consumption patterns. Fried potato products, bread, biscuits, breakfast cereals, and coffee contribute differently to exposure across population groups. Whether foods with high AA concentrations constitute major sources of total AA intake also depends on consumption frequency and portion size. Raw-material characteristics, formulation, storage, and processing conditions likewise affect AA concentrations in foods. For example, a survey of 20 commercial potato chip products found AA concentrations ranging from 93.51 to 1,777.96 μg/kg, with 10% of the samples exceeding the European Union benchmark level of 750 μg/kg (27). Consequently, exposure estimates based on mean AA concentrations for food categories may obscure variation among products or production batches (5).

Epidemiological studies assessing the association between dietary AA exposure and cancer have yielded inconsistent results. Uncertainty in dietary exposure assessment and potential confounding by smoking and other lifestyle factors further complicate their interpretation (28). Accordingly, toxicological hazards identified in experimental studies should be distinguished from the likelihood of adverse health outcomes at specific exposure levels; inconsistent epidemiological findings should not be interpreted as evidence of no risk.

For mitigation studies, lowering AA concentrations in foods and reducing population health risk are related but distinct outcomes. When initial AA concentrations differ, the same percentage reduction may correspond to different absolute decreases in concentration. The extent to which lower AA concentrations in a food reduce dietary exposure also depends on that food’s contribution to total dietary AA intake. Matrix-specific differences in precursor accessibility, mass transfer behavior, and thermal history further limit the direct extrapolation of mitigation results across food systems. Therefore, product-level reductions in AA alone cannot establish the magnitude of population-level health benefits; their health significance must be interpreted in light of actual exposure levels and the associated uncertainties.

## 3 Mechanistic basis and intervention targets for acrylamide mitigation

### 3.1 Major and auxiliary formation pathways

The reaction between asparagine and reducing sugars is the principal pathway for AA formation in potato- and cereal-based foods (29). Table 1 identifies asparagine and reducing sugars as the key determinants of this route and lists precursor removal, asparaginase treatment, and acidification as corresponding control targets. Heating studies in model systems have confirmed that these precursors can produce AA via the Maillard reaction (4, 30). As illustrated in Figure 2A, during the initial stage of the reaction, the α-amino group of asparagine condenses with the carbonyl group of reducing sugars, such as glucose or fructose, to form an unstable Schiff base (31). The decarboxylated Schiff base intermediate can form AA through direct elimination or undergo hydrolysis to yield 3-aminopropionamide (3-APA), which is subsequently converted to AA through deamination (29). The same reaction network also drives Maillard browning and the development of characteristic aromas and flavors (32). Mitigation strategies must therefore suppress AA formation without compromising the sensory quality developed during thermal processing (33, 34).

**Table 1:** Major formation pathways, influencing factors, and control targets for acrylamide in foods.

Formation pathway or influencing factor	Key determinants	Effect on AA formation	Representative food matrices	Potential control targets	Reference
Dominant asparagine–reducing sugar pathway	Asparagine and reducing sugars	Maillard reaction via Schiff base and 3-APA	Potato and cereal products	Precursor reduction, asparaginase, acidification	(3)
Acrolein-associated pathway in heated model systems	Acrolein or acrylic acid with ammonia	AA formation in heated model reactions; contribution in foods unquantified	No food matrix tested (model systems only)	Oil oxidation control (requires food-system validation)	(36)
Raw-material precursor levels and ratios	Precursor content, cultivar, and storage	Determines formation potential and limiting precursor	Potatoes, cereals, and coffee	Select low-precursor materials; optimize storage	(47)
Calcium salts in cereal formulation	Calcium salt type and dose (CaCl₂ or CaCO₃)	Lower measured AA at selected doses under the tested baking conditions	Wholemeal wheat cookies	Optimize calcium-salt addition while assessing product quality	(208)
Frying time–temperature history	Frying temperature, duration, and moisture loss	AA varied with frying conditions and was correlated with moisture content	Fried potato slices	Adjust frying conditions and verify final moisture and AA	(209)

AA, acrylamide; 3-APA, 3-aminopropionamide.

**Figure 2:**
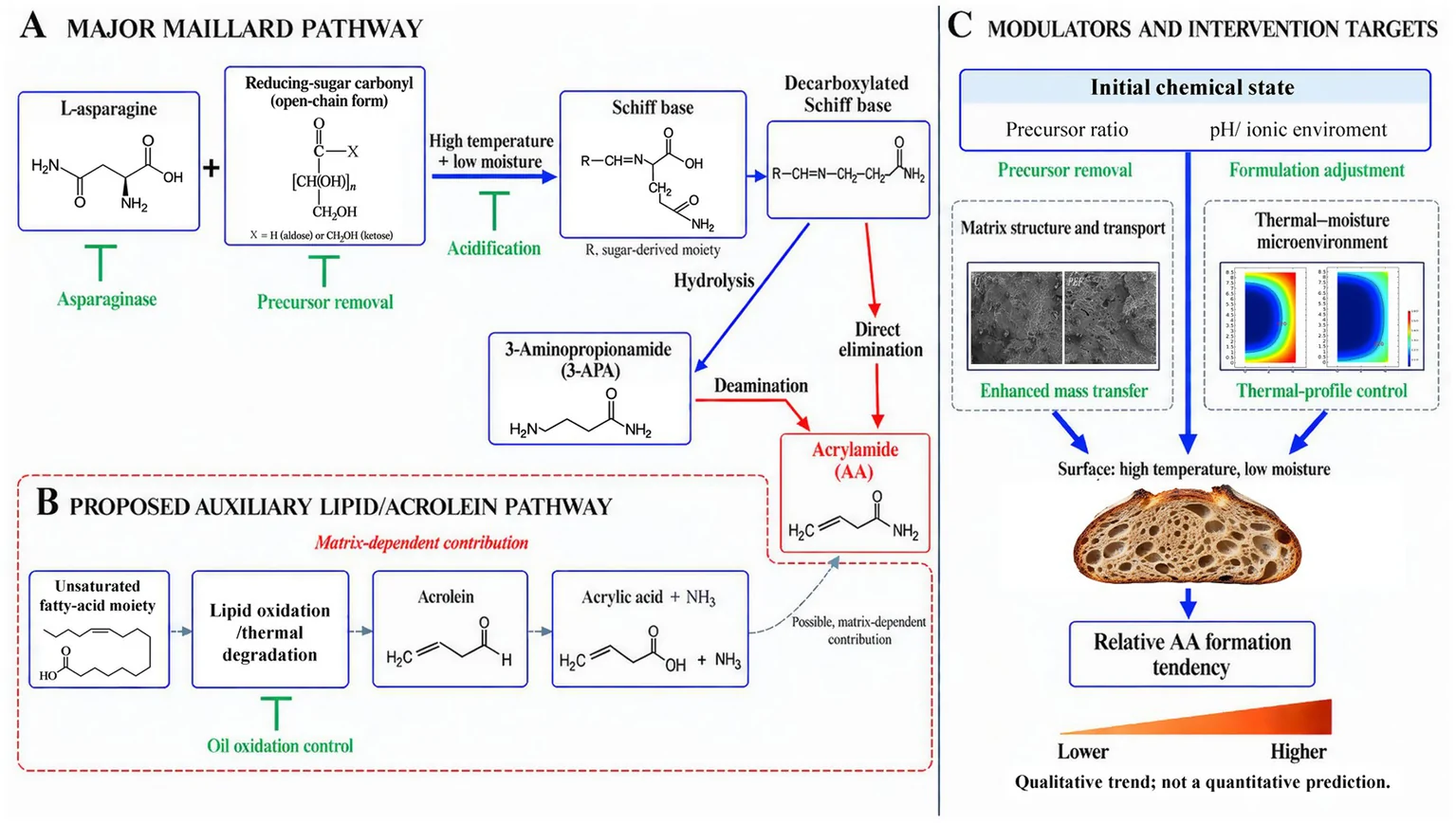
Major **(A)** and proposed auxiliary **(B)** pathways of acrylamide formation in thermally processed foods, with the principal modulators and intervention targets **(C)**. The lower-to-higher scale in panel C represents a qualitative trend rather than a quantitative prediction. Representative SEM images showing untreated and PEF-treated sweet-potato microstructures were adapted from Figure 8a,c of Liu et al. (197), while the model-derived temperature distributions comparing frying without and with a temperature-step approach were adapted from Figure 9a,b of Chan (205), both under the terms of the Creative Commons Attribution License (CC BY 4.0). Cropping, relabeling, and layout adjustments were performed for consistency. AA, acrylamide; 3-APA, 3-aminopropionamide; PEF, pulsed electric field.

In addition to the primary route, AA can also be generated through an acrolein-mediated pathway (35, 36). Figure 2B further shows that, in lipid-rich foods, the oxidation and thermal degradation of triacylglycerols (triglycerides) and unsaturated fatty acids can produce acrolein. Acrolein can subsequently be oxidized to acrylic acid, which may then react with ammonia and contribute to AA formation (7). The relative contribution of this pathway depends on the extent of lipid oxidation, ammonia availability, matrix composition, and thermal processing conditions. Accordingly, Table 1 lists control of lipid oxidation and thermal load as corresponding intervention targets. Controlling these factors may provide complementary mitigation effects but cannot replace control of the primary precursors, particularly asparagine and reducing sugars (37).

The dominant pathway of AA formation in a food determines the most appropriate point of intervention (38, 39). Controlling this pathway requires coordinated management of both asparagine and reducing sugars (40), whereas the acrolein-mediated route identifies lipid oxidation and excessive thermal degradation as supplementary targets. Because several formation pathways may operate simultaneously and interact, reducing a single precursor may not completely prevent AA formation. Effective mitigation should therefore target the key limiting factor within the specific product and processing system.

### 3.2 Precursor levels and raw-material factors

The concentrations and relative proportions of free asparagine and reducing sugars in raw materials largely determine the initial potential for AA formation (41, 42). Asparagine provides the carbon skeleton and nitrogen source of AA, while reducing sugars such as glucose and fructose supply reactive carbonyl groups (43–45). Although sucrose is a non-reducing sugar, its hydrolysis under acidic or thermal processing conditions releases glucose and fructose, thereby increasing its indirect contribution to AA formation (31). Higher precursor concentrations generally favor AA production, but the extent of formation ultimately depends on which reactant is limiting (29). The precursor ratio shown in Figure 2C illustrates how reactant balance modulates the extent of AA formation.

The limiting reactant varies with the raw material. Potato tubers generally contain abundant free asparagine, making reducing sugars the limiting components in many cases (8, 46). Under certain cultivar-specific or processing conditions, however, asparagine may instead become limiting (47). Kinetic studies further show that the rate of AA formation depends on the fructose-to-glucose ratio and the proportion of asparagine in the free amino acid pool (9). The concentration of a single precursor, considered in isolation, is therefore insufficient to predict the final amount of AA formed across different food categories.

Cultivar, maturity, growing environment, and storage conditions can substantially alter the carbohydrate and amino acid composition of raw materials (46, 48). Potato cultivars differ markedly in their concentrations of reducing sugars and asparagine, and even the same cultivar may respond differently to growing conditions, harvest time, and storage environment (47). Nitrogen fertilization affects amino acid synthesis, while sulfur deficiency can markedly increase free asparagine in cereals and thereby raise their potential for AA formation during baking (49, 50). Drought and temperature stress can likewise disrupt normal carbon and nitrogen metabolism in plants (51, 52). After harvest, excessively low storage temperatures induce cold-induced sweetening in potatoes, leading to the accumulation of glucose and fructose and substantially increasing AA formation during subsequent frying or baking (53). Cultivar, maturity, moisture content, and storage conditions exert similar influences in cereals and coffee, although the magnitude and direction of these effects depend on the raw material and the subsequent processing method. Table 1 relates precursor levels, cultivar, and storage conditions to AA formation potential and the identity of the limiting precursor, and identifies the selection of low-precursor raw materials and optimization of storage conditions as potential mitigation targets.

### 3.3 Food-matrix and thermal-process factors

Precursor composition alone cannot fully explain AA formation because the food matrix determines whether reactants can migrate, come into contact, and react. Figure 2C summarizes the constraints imposed by food-matrix microstructure on precursor migration and contact, and identifies enhanced mass transfer as an intervention approach to facilitate precursor removal. Starch gelatinization, protein denaturation, cell-wall disruption, and changes in porosity alter the movement of asparagine, reducing sugars, and water (54). During heating, potato slices, bread, and biscuits develop a dry surface crust (55, 56). The migration and local concentration of precursors at surfaces and edges help explain why AA concentrations are often substantially higher in these regions than in the interior (6). Proteins and other free amino acids can compete with asparagine for reactive carbonyl compounds, thereby redirecting parts of the Maillard reaction network (57–59). In lipid-containing foods, reactive carbonyls generated by lipid oxidation may make an additional contribution to AA formation, although the extent of this contribution depends on lipid composition, oxidation state, and matrix structure (36, 60).

In addition to the effects of matrix structure and mass transfer described above, Figure 2C and Table 1 identify pH and ionic composition as important factors regulating precursor reactivity within the local reaction microenvironment. Acidification protonates the amino group of asparagine, making Schiff base formation less favorable, whereas neutral or mildly alkaline conditions generally promote the Maillard reaction. Cations such as calcium and magnesium can interact with asparagine or reaction intermediates. Their effects are not constant but vary with ion type, concentration, and the composition of the surrounding food matrix. Accordingly, Table 1 identifies formulation adjustment and precursor removal as corresponding control targets in potato products, baked goods, and composite foods.

Thermal history describes the temperature profile experienced by a food over time, including the heating rate, peak temperature, and residence time at elevated temperature (61). Figure 2C identifies thermal history, moisture migration, and the mode of heat transfer as interrelated regulatory factors that jointly determine the dynamic balance between AA formation and degradation (62, 63). Higher temperatures accelerate the condensation of asparagine with reducing sugars, decarboxylation of the Schiff base, and deamination of 3-aminopropionamide (55). When other conditions are comparable, frying, baking, or roasting at higher temperatures therefore generally increases AA formation (64). Accordingly, Table 1 identifies limiting high-temperature, low-moisture exposure as the corresponding control target for this class of factors.

## 4 Analytical verification and process monitoring

### 4.1 Sample preparation and matrix-effect control

The low molecular weight, high polarity, low volatility, and weak ultraviolet absorption of AA make its accurate quantification in foods particularly challenging. At the same time, sugars, amino acids, proteins, lipids, pigments, and other Maillard reaction products may be co-extracted with AA, co-elute during chromatographic separation, or cause ion suppression in mass spectrometric analysis (18, 65). Sample preparation can therefore influence the result as much as instrumental detection.

Most analytical workflows, as illustrated in Figure 3A, begin with sample homogenization and the addition of an isotopically labeled internal standard before extraction with water or an aqueous–organic solvent mixture (66). The extent of subsequent cleanup depends on the composition of the food matrix. For some starch-rich foods, aqueous extraction followed by centrifugation and filtration may be sufficient; by contrast, high-fat or high-protein matrices may additionally require defatting, protein precipitation, or solid-phase extraction (SPE) to remove interfering components. Coffee, cocoa, seasonings, and similarly complex food matrices also require more selective cleanup because their extracts contain large amounts of both polar and nonpolar matrix components (18). These matrix-dependent adaptations are reflected in the representative workflows in Table 2, including defatting, Carrez clarification, dispersive solid-phase extraction, and derivatization.

**Figure 3:**
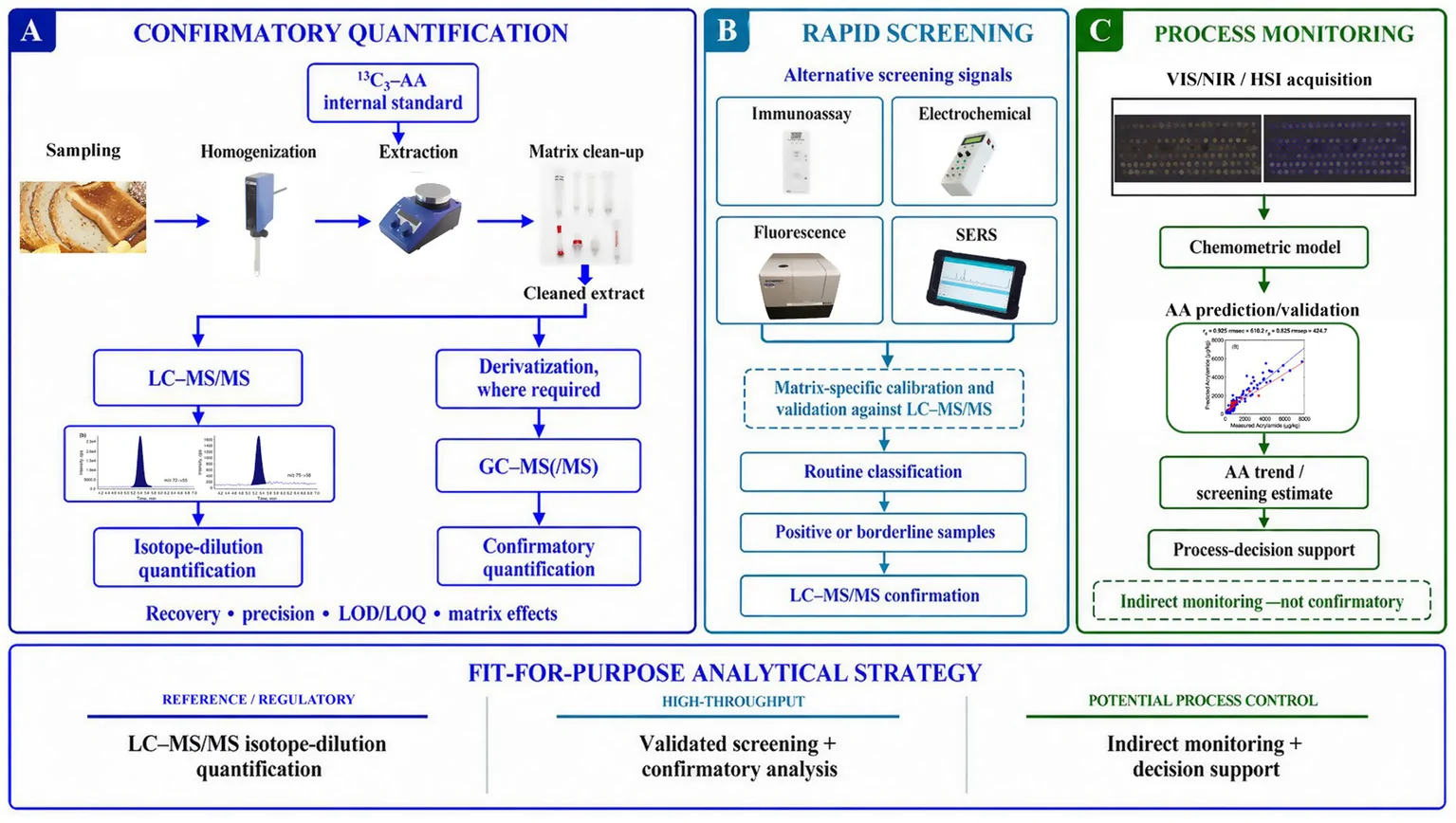
Fit-for-purpose analytical strategies for acrylamide: confirmatory quantification **(A)**, rapid screening **(B)**, and process monitoring **(C)**. In panel A, representative paired multiple-reaction-monitoring chromatograms of acrylamide (AA) and ^13^C₃-AA in a potato-chip matrix were adapted from Figure 1b of Lee et al. (206) under the terms of the Creative Commons Attribution License (CC BY). In panel C, representative hyperspectral-imaging sample images and segmentation results were adapted from Figure 1 of Bruno et al. (93), and the observed-versus-predicted AA validation plot was adapted from Figure 4B of the same reference; both adaptations were made under the terms of the Creative Commons Attribution License (CC BY 4.0). Cropping, relabeling, and layout adjustments were performed for visual consistency. Representative images in panel B illustrate four alternative screening methods: immunoassays, electrochemical sensors, fluorescence-based methods, and SERS. These images are illustrative and do not represent quantitative results. AA, acrylamide; HSI, hyperspectral imaging; LC–MS/MS, liquid chromatography–tandem mass spectrometry; LOD, limit of detection; LOQ, limit of quantification; MRM, multiple reaction monitoring; NIR, near-infrared; SERS, surface-enhanced Raman spectroscopy.

**Table 2:** Comparison of representative methods for acrylamide analysis, with food-matrix and sample-preparation context.

Method category	Core analytical principle	Food matrix/validation material	Sample preparation or measurement context	Representative analytical performance	Main advantages	Main limitations	Primary application	Reference
Confirmatory quantification								
LC–MS/MS or UHPLC–MS/MS	Isotope-dilution MRM	Infant formula: 9–90 μg/kg spikes; matrix CRM;	Water; 0.1% formic-acid acetonitrile; n-hexane; MgSO4/NaCl; basic alumina dSPE	LOQ, 0.90 μg/kg; recovery, 103–105%; RSD, 1.3–1.6%	Sensitive and selective	High cost; matrix effects	Confirmatory quantification	(70)
GC–MS or GC–MS/MS	GC separation with MS detection	Food matrices	Non-aqueous flavanol derivatization (40 °C, 1 min)	LOD, 0.7 μg/kg; RSD, 2.3–6.1%	Strong separation and identification	Derivatization; potential analyte loss	Confirmatory analysis	(65)
Rapid screening and preliminary classification								
HPLC–UV/DAD	LC separation with UV/DAD detection	French fries; bakery and branded biscuits; blank-water recovery	Defatting; water extraction; Carrez I/II; filtration	LOD/LOQ, 3.733/11.045 ng/μL; recovery, 100.6%	Accessible and inexpensive	Weak AA absorbance; coelution	Routine determination	(73)
Electrochemical and molecularly imprinted sensors	Electrochemical signal transduction	Diverse matrices and spiked systems	Platform-specific preparation	Performance varies by sensor design	Rapid and miniaturizable	Fouling, selectivity, and stability	Rapid screening	(76)
Fluorescent and aptamer-based nanosensors	Aptamer-amplified fluorescence	Spiked coffee and potato chips; QC potato chips and cookies	Water dispersion; sonication; n-hexane; Carrez cleanup; 500-fold dilution; LOD established by standard-solution calibration	LOD, 1.802 pM	Sensitive and rapid	Probe complexity; optical interference	High-sensitivity detection	(210)
Process monitoring and decision support								
Near-infrared, FTIR, and hyperspectral imaging	Spectral modelling by PLS/PLSR	Ground potato crisps	Direct VIS/NIR HSI measurement; six-wavelength PLSR model for the low-AA subset (<3,000 μg/kg)	PLSR prediction: *r* = 0.89	Rapid and nondestructive	Calibration-dependent	Process screening and early warning	(93)

AA, acrylamide; CRM, certified reference material; CV, coefficient of variation; DAD, diode-array detection; dSPE, dispersive solid-phase extraction; ECIA, electrochemical immunoassay; Exo III, exonuclease III; GC–MS, gas chromatography–mass spectrometry; HPLC, high-performance liquid chromatography; HSI, hyperspectral imaging; ic-ELISA, indirect competitive enzyme-linked immunosorbent assay; LC–MS/MS, liquid chromatography–tandem mass spectrometry; LOD, limit of detection; LOQ, limit of quantification; MIP, molecularly imprinted polymer; MRM, multiple reaction monitoring; NIR, near-infrared; PLSR, partial least-squares regression; RSD, relative standard deviation; UHPLC–MS/MS, ultra-high-performance liquid chromatography–tandem mass spectrometry; VIS, visible.

Insufficient cleanup has been reported to overestimate AA concentrations in cocoa by up to 20-fold, whereas the co-elution of N-acetyl-β-alanine may cause AA concentrations in coffee to be overestimated by approximately 40% (66). For coffee and coffee products, single-step mixed-mode SPE combined with bromination derivatization can improve analyte retention and robustness against matrix interference, yielding recoveries of 99.3–102.2% (67). However, this analytical performance was achieved using a method specifically developed for the coffee matrix and should not be assumed to apply to other food systems without modification.

The high water solubility of AA makes selective purification of aqueous extracts a persistent analytical bottleneck (18). Modified quick, easy, cheap, effective, rugged, and safe (QuEChERS) methods, molecularly imprinted sorbents, microextraction techniques, and other low-solvent-consumption approaches can reduce matrix interference and solvent use (68); however, no single sample-preparation method is universally applicable to all food matrices (18, 67). Stable isotope dilution can compensate for some analyte losses during extraction and variations in instrumental response, but in the presence of co-eluting interferences, it cannot replace adequate chromatographic selectivity and matrix-specific validation (66, 69). Therefore, the suitability of a sample-preparation method should be demonstrated for each food matrix using data on recovery, precision, selectivity, and matrix effects, rather than judged solely on extraction efficiency.

### 4.2 Confirmatory chromatographic–mass spectrometric methods

In the confirmatory quantitative workflow shown in Figure 3A, liquid chromatography–tandem mass spectrometry (LC–MS/MS) and ultra-high-performance liquid chromatography–tandem mass spectrometry (UHPLC–MS/MS) are the principal methods used to quantify AA in foods (65). LC–MS/MS generally enables direct analysis without derivatization; however, for certain applications, derivatization may be used to improve the chromatographic retention of the analyte and reduce matrix interference (67). Data are subsequently acquired in multiple reaction monitoring (MRM) mode by monitoring selected precursor-to-product ion transitions (70).

Positive-mode electrospray ionization (ESI) is the ionization technique most commonly used for the LC–MS/MS determination of AA. When ESI does not provide sufficient response or stability under the selected chromatographic conditions or in the target food matrix, atmospheric-pressure chemical ionization (APCI) can serve as an alternative. The analytical performance of both ion sources is affected by mobile-phase composition, analyte form, co-eluting matrix components, and ion-source parameters. The more suitable ion source for a target food matrix should be selected by comprehensively comparing the signal intensity, matrix effects, repeatability, and method robustness achieved with ESI and APCI (17, 65).

Because AA is highly polar, its retention on conventional reversed-phase columns may be weak, increasing the likelihood of co-elution with early-eluting polar compounds; chromatographic selectivity is therefore particularly important. Existing methods commonly use reversed-phase columns compatible with highly aqueous mobile-phase conditions, columns with amide stationary phases, or porous graphitized carbon columns to improve AA retention and separation (17, 65). A recent method developed for powdered infant formula compared three columns: BEH Amide, HSS T3, and Atlantis dC18. The retention times of AA on the first two columns were approximately 1.7 and 2.2 min, respectively. These short retention times may increase matrix interference, whereas the Atlantis dC18 column extended the retention time to approximately 4.0 min and was therefore selected for subsequent AA analysis (70). This comparison indicates that column selection should not be based solely on analysis time and peak shape, but should also consider analyte retention, separation from known interferents, and matrix effects. The smaller particle sizes and higher operating pressures used in UHPLC–MS/MS shorten analysis time and improve chromatographic peak shape; however, faster separation cannot compensate for insufficient retention or inadequate resolution of matrix interferences (66).

Adding isotopically labeled AA as an internal standard helps correct for recovery losses during sample preparation and variations in instrument response (69), but cannot eliminate inadequately resolved co-eluting interferences. To reduce the risk of bias in AA quantification caused by such interference, method performance should be validated in the target food matrix (66). As shown in Figure 3A, method validation should include recovery, precision, limits of detection and quantification, and matrix effects, and should further evaluate selectivity, calibration linearity and range, trueness, carryover, and analyte stability. Precision can be assessed in terms of repeatability and intermediate precision.

A single-laboratory validation of the AOAC First Action method, completed in 2024, covered coffee, cereals, infant foods, cocoa, dry pet food, potato products, vegetable chips, biscuits, tea, nuts, and spices. The reported recoveries ranged from 97 to 108%, while the relative standard deviations for both repeatability and intermediate precision were below 12% (66). By contrast, the powdered-infant-formula method included in Table 2 combined isotope dilution with matrix-specific cleanup and achieved recoveries of 103–105%, relative standard deviations below 1.6%, and a limit of quantification of 0.90 μg/kg; method accuracy was further evaluated using a certified reference material (70). These results demonstrate the analytical capabilities of the respective methods within their validated matrices, but should not be assumed to directly represent performance in other food systems without additional validation.

Overall, LC–MS/MS and UHPLC–MS/MS provide the specificity and sensitivity required for the confirmatory determination of trace AA in complex food extracts (70). Their principal limitations are the high costs of instrument acquisition and maintenance and the need for specialized expertise in instrument operation and data interpretation (18).

Gas chromatography–tandem mass spectrometry (GC–MS/MS) is another established and reliable platform for AA analysis (69, 71). Because AA is highly polar and has low volatility, GC-based workflows generally use bromination, silylation, or other derivatization procedures to improve analyte volatility and thermal stability. Together, GC–MS/MS and LC–MS/MS constitute the two principal mass spectrometric approaches for determining AA in foods (17). GC–MS/MS provides strong chromatographic separation, and the resulting mass spectra can be compared with established spectral libraries. Derivatization, however, lengthens the workflow and may introduce errors through incomplete reactions, impurities in derivatization reagents, or sample-transfer steps. LC–MS/MS usually offers a simpler workflow without derivatization, although its ionization efficiency remains susceptible to matrix-induced suppression or enhancement (17, 65).

### 4.3 Conventional and rapid screening methods

High-performance liquid chromatography with ultraviolet detection (HPLC–UV) and high-performance liquid chromatography with diode-array detection (HPLC–DAD) are less expensive and more widely available than mass spectrometric platforms (72). Because AA absorbs ultraviolet radiation only weakly, these methods generally use short detection wavelengths or derivatization to increase the analytical signal. Co-eluting compounds may also absorb in the same spectral region, however, reducing method selectivity (65, 73). HPLC–UV is therefore most suitable for relatively simple matrices, samples containing higher AA concentrations, or preliminary screening (72). Its suitability should be assessed from recovery, precision, linear range, limit of detection (LOD), limit of quantification (LOQ), matrix effects, and the effectiveness of sample clean-up.

Immunoassays rely on antibodies or nanobodies to recognize AA derivatives and convert antigen–antibody binding into measurable optical or electrochemical signals for indirect determination. Because AA is a small-molecule hapten, it must first be conjugated to a carrier protein or reacted with a derivatizing reagent to produce a recognizable antigenic structure. For example, the Nb-7E nanobody, which recognizes xanthene-based AA derivatives, has been applied in an indirect competitive enzyme-linked immunosorbent assay (ic-ELISA) and an electrochemical immunoassay (74). The respective LODs were 0.089 and 0.033 μg/mL, and the electrochemical format showed good linearity over 0.39–50.0 μg/mL. Spiked biscuit and potato-chip samples yielded recoveries of 88.29–111.76%, with results closely agreeing with those obtained by UHPLC–MS/MS. These findings indicate that nanobody-based assays combine specific recognition with signal amplification and can serve as complementary screening tools, although they cannot yet replace chromatography–mass spectrometry for confirmatory analysis.

Electrochemical sensors quantify AA by measuring changes in current, potential, or impedance arising from its interaction with hemoglobin, enzymes, aptamers, or molecularly imprinted recognition sites (19). Carbon nanotubes, graphene, gold nanoparticles, and metal–organic frameworks (MOFs) increase the specific surface area of the electrode and facilitate electron transfer, thereby amplifying the analytical signal (75, 76). Hemoglobin-based biosensors have shown high analytical sensitivity, which can be further improved by modification with carbon nanotubes or gold nanoparticles (19). Molecularly imprinted polymers (MIPs) provide an alternative recognition strategy by creating cavities that complement AA in size, shape, and functional-group arrangement. Their chemical stability and reusability are advantageous, but residual template molecules and cross-reactivity with structurally similar small compounds remain concerns.

Fluorescence assays detect AA through probe responses such as fluorescence enhancement, quenching, energy transfer, or conformational change. Carbon dots, semiconductor quantum dots, metal nanoclusters, and metal–organic frameworks (MOFs) can be combined with aptamers, molecular imprinting, or derivatization to improve recognition specificity (77–79). Surface-enhanced Raman spectroscopy (SERS) instead uses the localized electromagnetic fields generated by gold, silver, or related nanostructures to amplify the characteristic Raman scattering signals of AA and its derivatives (80, 81). Both approaches permit rapid sample analysis, but food color, turbidity, ionic strength, and coexisting matrix components can substantially interfere with analytical performance.

The examples described above indicate that immunoassays can achieve low detection limits and satisfactory spike recoveries, although the scope of the supporting evidence remains to be clearly defined. For example, the Nb-7E nanobody method yielded results that were in relatively good agreement with those obtained by UHPLC–MS/MS for spiked biscuit and potato-chip samples, supporting its feasibility for screening these two food matrices (74). Because the available validation is based primarily on spiked samples, these findings do not establish whether the method is equally applicable to samples in which AA is generated during processing or to other food matrices. Moreover, reproducibility across reagent lots and laboratories has not been systematically validated and requires further evaluation.

For electrochemical, molecularly imprinted, fluorescence, and surface-enhanced Raman spectroscopy (SERS) sensors, analytical performance may be affected not only by matrix interference arising from food color, turbidity, ionic strength, and coexisting components, but also by batch-to-batch variation in sensing materials and detection interfaces. The response of electrochemical sensors may depend on electrode material composition, nanomaterial loading, and electrode surface condition, whereas the performance of molecularly imprinted polymers depends on polymerization conditions and the extent of template removal. Variations in the particle size and degree of surface functionalization of fluorescent probes and SERS substrates, as well as in substrate uniformity, may also alter background signals, sensitivity, calibration slopes, and measurement repeatability (19, 76, 78, 80). These methods therefore require batch-level quality control and matrix-specific calibration, and analytical performance established using standard solutions or a single spiked food matrix should not be directly extrapolated to other food systems.

These limitations define the scope of application of the rapid methods shown in Figure 3B. Immunoassays and sensors can be used for rapid sample screening and preliminary classification, but each method requires matrix-specific validation; positive, borderline, or anomalous results should be confirmed using a validated LC–MS/MS method. At present, the standardization, interlaboratory reproducibility, and regulatory acceptance of rapid methods as stand-alone confirmatory methods remain limited. Consistent with this intended role, AOAC SMPR 2022.006 specifies that quantitative analysis of AA for monitoring and surveillance should employ techniques that combine chromatographic separation with mass spectrometric detection (82).

### 4.4 Spectroscopic process monitoring and decision support

Process monitoring may target AA itself, its precursors, or process variables that characterize the conditions under which it forms (83, 84). Because most quantitative workflows require extraction and clean-up, direct real-time online measurement remains difficult (85). Processing-control studies therefore commonly monitor reducing sugars, free asparagine, temperature, moisture content, water activity, and color as indirect indicators (86).

Near-infrared spectroscopy (NIR) uses molecular vibrational absorption to provide compositional information on moisture (87), carbohydrates, and related constituents (88, 89). Hyperspectral imaging (HSI) adds spatial information, allowing differences in surface browning or moisture status to be mapped across a sample, while machine vision can record changes in lightness and color-difference parameters (90). None of these non-destructive techniques is equivalent to direct AA quantification. Their correlations with AA concentration may shift with changes in raw-material composition, food-matrix structure, and processing method (91).

The use of near-infrared spectroscopy (NIR) in industrial production to monitor food composition and processing status should be distinguished from the online prediction of AA. A study using an online visible/near-infrared (VIS/NIR) line-scan system to inspect potato chips reported a correlation coefficient of 0.83 between predicted and reference AA values and a mean prediction error of 266 μg/kg, indicating that the system could identify samples with abnormally high AA concentrations (92). This study supports the technical feasibility of online AA screening but does not establish that such systems have been routinely deployed in industry or integrated into closed-loop process control. Other studies using NIR and hyperspectral imaging (HSI) to predict AA have primarily developed product-specific calibration models under defined instrumental and processing conditions (85, 88, 93). The available evidence is therefore insufficient to demonstrate that online NIR systems for AA prediction are routinely used on commercial food production lines.

These models require recalibration and external validation against measurements obtained using reference quantitative methods when transferred across varieties, production seasons, factories, processing lines, and spectroscopic instruments (94). As illustrated in Figure 3C, NIR, HSI, machine vision, and conventional process variables are better suited for predicting trends in AA levels, providing early warnings, and supporting processing decisions. Following adequate validation, these approaches may further support feedback control; however, they cannot replace chromatographic–mass spectrometric methods for confirmatory or regulatory quantitative analysis.

### 4.5 Guide to method selection

Method selection depends on the analytical purpose, food-matrix characteristics, and the level of reliability required when results are used for decision-making; it should not be based solely on comparisons of limits of detection or quantification. Figure 3 distinguishes three practical applications: chromatography–mass spectrometry can provide reliable direct confirmation and quantification; immunoassays and sensor-based methods are more suitable for rapid sample screening; and spectroscopic techniques combined with process-variable monitoring provide indirect information for trend assessment and processing decisions. Table 2 lists the analytical performance of representative methods alongside the relevant food matrices and sample-preparation conditions, enabling recovery data and limits of detection and quantification to be interpreted in the context of specific method-validation conditions rather than compared in isolation.

## 5 Mechanism-guided mitigation and control technologies

AA mitigation can be directed at four principal intervention points: reducing precursor levels, controlling the Maillard reaction microenvironment, enhancing mass transfer, and shortening the high-temperature, low-moisture reaction stage (7). Each technology acts at a different point. Raw-material selection, soaking, and blanching reduce the initial precursor load before heating. Asparaginase hydrolysis and fermentation consume asparagine or reducing sugars, whereas acidification and formulation adjustment lower chemical reactivity by altering the ionization state of reactive groups. Pulsed electric field (PEF), ultrasound, and high-pressure processing (HPP) mainly modify cellular and tissue structures, promoting precursor leaching or the penetration and diffusion of enzyme solutions. Cold atmospheric plasma (CAP) acts through reactive oxygen and nitrogen species (ROS/RNS) and surface micro-etching or modification, while plasma-activated water (PAW) delivers reactive species through the aqueous phase. Vacuum processing and emerging physical-field or alternative-heating technologies alter dehydration rates, energy distribution, moisture migration, and thermal history, thereby shifting the balance between AA formation and degradation (95, 96).

### 5.1 Pulsed electric field-assisted mitigation

Pulsed electric field (PEF) treatment exposes foods to high-voltage electric pulses lasting from microseconds to milliseconds. When the induced transmembrane potential exceeds the critical threshold of the cell membrane, reversible or irreversible electroporation occurs, substantially increasing the permeability of plant tissues (97). In intact potato and sweet potato tissues, this structural modification promotes the release of intracellular glucose, fructose, and free asparagine, facilitating the removal of these precursors during subsequent washing, soaking, or blanching. As shown in Figure 4A, PEF promotes precursor release and, when combined with washing or blanching, facilitates the leaching of free precursors (98, 99). Electroporation can also soften plant tissue, accelerate internal moisture migration, and shorten subsequent frying or drying times (100). PEF is therefore a physical pretreatment based on microstructural modification and enhanced mass transfer, rather than a chemical process that directly degrades AA after it has formed.

**Figure 4:**
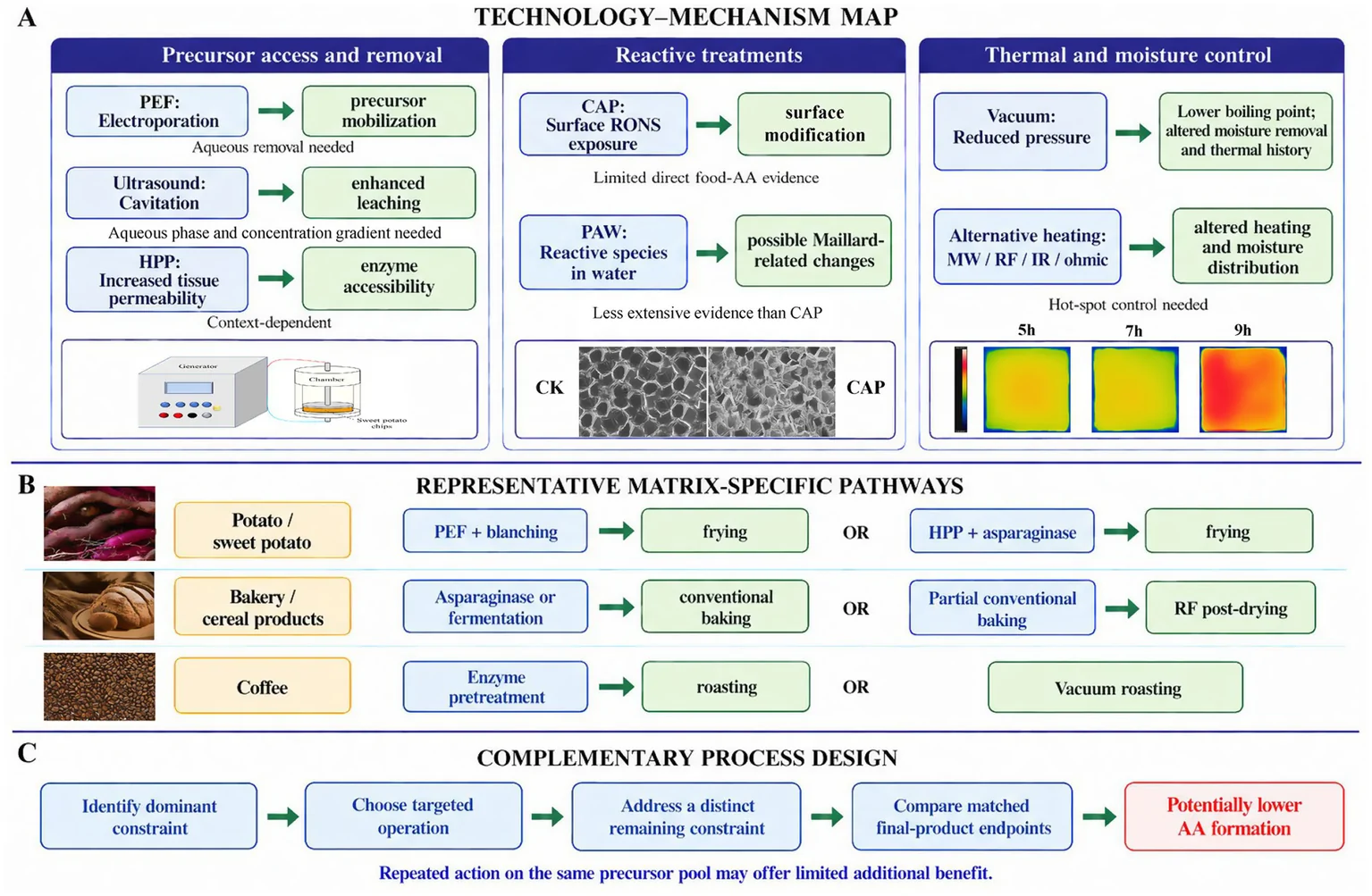
Emerging mitigation technologies for acrylamide (AA): mechanism mapping **(A)**, matrix-specific pathways **(B)**, and complementary process design **(C)**. The PEF treatment setup was adapted from Figure 1 of Liu et al. (197) under CC BY 4.0; control (CK) and CAP-treated potato-strip SEM micrographs (CAP: 60 kV, 15 min) were adapted from Figures 1A,D of Nateghi et al. (12) under the CC BY license; and experimental infrared thermograms at 5, 7, and 9 h were adapted from Figure 4 of Chen et al. (207) under CC BY 4.0. Cropping, relabeling, and layout adjustments were made for visual consistency. “OR” indicates alternative pathways and does not imply demonstrated synergy. AA, acrylamide; CAP, cold atmospheric plasma; CK, untreated control; HPP, high-pressure processing; IR, infrared; MW, microwave; PAW, plasma-activated water; PEF, pulsed electric field; RF, radio frequency; RONS, reactive oxygen and nitrogen species; SEM, scanning electron microscopy.

The effects of PEF depend on electric field strength, pulse width and number, specific energy input, temperature, system conductivity, and tissue structure (101, 102). Lamoka potatoes treated at 1.0 kV/cm with a specific energy input of 0.75–1.5 kJ/kg were used to evaluate these effects (10). At the higher treatment intensity, glucose, fructose, and free asparagine concentrations decreased by 30.9%, 19.5%, and 45.7%, respectively. Subsequent frying time was shortened by approximately 10%, final AA content decreased by about 29%, and oil content was also reduced. The greater reduction in free asparagine may have been observed under a specific PEF–washing protocol. PEF promotes the overall mass transfer of water-soluble precursors, whereas the actual reductions in individual precursors remain dependent on the food matrix, PEF conditions, and subsequent washing conditions (103). These findings are consistent with a multistage physical mechanism in which electroporation alters precursor leaching, migration, and dehydration kinetics during subsequent processing. They should not be interpreted as evidence that PEF directly degrades AA through a single chemical reaction.

For potatoes and sweet potatoes with denser tissue structures, Figure 4B outlines a common PEF-assisted treatment strategy in which PEF is applied as a pretreatment together with washing or blanching to remove released precursors, followed by vacuum treatment or optimized frying to regulate the subsequent thermal process. Although the overall treatment pathway is similar across food matrices, the PEF intensity required for effective pretreatment may vary considerably with raw-material characteristics (104). In the production of thick-cut sweet potato kettle chips, PEF was applied at an electric field strength of 1.0 kV/cm (105). At a specific energy input of 3.0 kJ/kg, AA content decreased from 0.668 to 0.370 μg/g, a reduction of 44.6%, while oil content fell by approximately 8.5%. At lower specific energy inputs, however, AA content did not change significantly. Differences between sweet potato and conventional potato in cellular architecture, dry-matter composition, and electrical conductivity affect both electroporation efficiency and precursor mass transfer. Insufficient energy input may fail to increase tissue permeability, whereas excessive treatment can intensify the loss of water-soluble solids, over-soften the tissue, or damage slice edges. Applied voltage or electric field strength alone is therefore insufficient to characterize and compare PEF processes. Specific energy input and the physical properties of the material must also be reported.

### 5.2 Plasma-based mitigation technologies

#### 5.2.1 Cold atmospheric plasma

Cold atmospheric plasma (CAP) contains reactive components such as electrons, ions, free radicals, excited molecules, and ultraviolet photons, allowing it to modify food-surface chemistry and microstructure at near-ambient temperatures (106). As shown in Figure 4A, CAP is positioned as a treatment approach targeting the modification of surface-associated precursors. Recent studies using free-amino-acid model systems further indicate that CAP treatment can generate hydrogen peroxide, ozone, peroxynitrite, nitrate, and nitrite, and induce the deamidation of free asparagine, together with oxidation, nitration, and nitrosation modifications (107). Deamidation converts free asparagine to aspartic acid, thereby reducing the concentration of the principal amino acid precursor available for AA formation, whereas oxidation and nitrosation alter the chemical forms of other free amino acids. Observations in these model systems suggest that CAP may influence AA formation by inducing precursor conversion, but they do not directly establish that this mechanism reduces AA concentrations in intact food systems.

Plasma exposure can also modify cell walls, starch-granule microstructure, and surface porosity, with subsequent effects on moisture removal and solute mass transfer (108). Recent work on potato starch further showed that moisture content altered the extent of plasma-induced surface etching, molecular-weight loss, and changes in ordered structure, indicating that the surrounding water content helps determine the structural response to CAP (109). Because these reactions occur mainly at the material surface, treatment effectiveness depends on the discharge-system configuration, working gas, discharge power, frequency, ambient humidity, sample distance, exposed surface area, and treatment time (110).

Before undergoing identical blanching and frying procedures, potato strips were pretreated for 5–15 min using a 60 kV dielectric barrier discharge (DBD) CAP system (12). After 15 min of treatment, asparagine decreased from 667.81 to 562.28 mg/100 g dry weight (DW), reducing sugars declined from 232.01 to 1,05.74 mg/100 g, and AA in the fried product fell from 1,807.35 to 346.86 μg/kg, corresponding to an 80.8% reduction. However, this study did not assess potential reaction by-products arising from CAP treatment. Oxidative conversion of the precursors, disruption of the microstructure during CAP treatment, and increased leaching during subsequent blanching may all have contributed to the observed decrease. The same study also found that 15 min of CAP produced the largest reduction in AA but not the lowest total oil content, which occurred after 5 min. Thus, the treatment duration selected for AA mitigation may not simultaneously optimize other product responses. Compared with PEF, however, CAP-mediated AA mitigation has been investigated in fewer studies and food matrices. Its low-temperature, surface-directed mode of action remains of interest, but the specific reaction intermediates, process reproducibility, and relative contributions of individual unit operations require further investigation.

#### 5.2.2 Plasma-activated water

Plasma-activated water (PAW) contains hydrogen peroxide, nitrite, nitrate, and other short- and long-lived reactive species generated through interactions between plasma and water at the gas–liquid interface. It can be applied to food materials by soaking, washing, or spraying (111). The resulting acidic and oxidative environment may alter the reactivity of asparagine and reducing sugars or inactivate endogenous enzymes. Compared with direct CAP treatment, liquid-phase contact provides more uniform exposure of the material. However, PAW composition changes with the working gas, discharge duration, source-water quality, initial pH, storage time, and application conditions. The reactive species also undergo spontaneous disproportionation and decay after generation.

A study of cookies reported that plasma-activated water (PAW) treatment reduced the levels of advanced glycation end products and methylimidazole, accompanied by decreases in free-radical signals and glyoxal and methylglyoxal levels (112). These results indicate that PAW can alter selected Maillard reaction–related processes in baked-food matrices and reduce the formation of heat-processing hazards. However, AA was not measured in this study; therefore, its findings cannot be used to determine the effects of PAW on AA precursors or final AA concentrations in food. When PAW is applied for AA mitigation, its effectiveness, potential reaction by-products, effects on product quality, and the stability of active components under industrial application conditions still require independent evaluation in specific food systems.

### 5.3 Ultrasound and high-pressure processing

#### 5.3.1 Ultrasound-assisted mass transfer

In liquid media, ultrasound generates cavitation bubbles whose formation, oscillation, and collapse produce shear forces, microjets, and localized turbulence (113–115). These mechanical and hydrodynamic effects reduce the thickness of the boundary layer at the solid–liquid interface and accelerate the leaching and transport of intracellular water-soluble compounds into the surrounding liquid (116–119). Because asparagine and reducing sugars are both water soluble, ultrasound is generally combined with soaking, washing, or hot-water blanching rather than used as a standalone method for AA control (120, 121).

Operating frequency and rated power alone do not fully characterize an ultrasonic treatment. The effective acoustic power delivered to the system, acoustic power density, treatment temperature and duration, ultrasonic-bath geometry, and liquid-to-solid ratio all influence cavitation intensity and mass-transfer efficiency (122, 123). Acoustic-field distributions also differ substantially among devices, so results obtained at the same rated power are not necessarily directly comparable.

When potato slices were sonicated for 30 min at 35 kHz, an acoustic power density of 95.2 W/kg, and 42 °C, AA content in the fried product was approximately 83.1% lower than in samples fried without pretreatment and about 50% lower than in samples soaked in water at the same temperature and for the same duration. The amount of reducing sugars leached from the tissue also increased by approximately 31% (11). The difference between the two comparisons indicates that the overall reduction reflected both warm-water soaking and the additional mass-transfer enhancement produced by ultrasonic cavitation.

In a preliminary experiment combining 2 min of ultrasound treatment at 1500 W and 100% amplitude with hot-water washing, the maximum reduction in AA reached 67.1% (124). This reduction was not consistently reproduced when the raw-material cultivar, storage conditions, or washing procedure changed, and hot-water washing then became the dominant step. These findings indicate that ultrasound is an auxiliary physical treatment that intensifies washing or extraction processes. Its effectiveness depends on the initial concentrations of precursors in the raw material and on whether the surrounding liquid can rapidly and continuously carry away solutes released from cellular tissues. The same principle applies to the PEF pretreatment discussed in Section 5.1. For physical pretreatments that act by increasing tissue permeability or enhancing mass transfer, precursor release can translate into net removal only when a concentration gradient is maintained between the food matrix and the external liquid phase.

#### 5.3.2 High-pressure processing for enhanced enzyme accessibility and mass transfer

High-pressure processing (HPP) applies uniform hydrostatic pressure and can alter cell-membrane structure, cell-wall conformation, and tissue porosity (125). HPP does not usually degrade AA directly or hydrolyze asparagine. Its main processing value lies in promoting the penetration and diffusion of water or externally added enzyme solutions into dense plant tissues (126).

Potato strips were immersed in a solution containing 10,000 asparaginase units (ASNU)/L and then held at 100–400 MPa for 5 min (127). The combined HPP and asparaginase treatment reduced AA content after frying by 26–47%, whereas pressure or enzyme treatment alone produced no marked reduction. This contrast is consistent with improved enzyme access under pressure, but it does not fully establish the mechanism or quantify the contribution of each operation to the combined reduction. The same microstructural changes may also cause excessive tissue softening or increase the loss of water-soluble solids. Practical process design must therefore consider pressure level, holding time, and tissue compactness together.

### 5.4 Vacuum and alternative heating technologies

#### 5.4.1 Vacuum frying, baking, and roasting

Vacuum frying, vacuum baking, and vacuum roasting reduce the system pressure and the boiling point of water, allowing moisture to be removed at lower product temperatures (13, 128, 129). Figure 4A illustrates how a lower boiling point alters the timing and temperature of moisture removal from food surfaces. Under high-moisture conditions, precursor dilution and evaporative cooling can suppress AA formation; as the surface gradually dries, precursors become locally concentrated as the temperature rises; and at very low moisture contents, molecular mobility and mass transfer are again constrained (62, 63). Moisture removal at lower temperatures slows the condensation reaction between asparagine and reducing sugars. Reduced pressure may also promote the loss of AA from the food matrix, although the relative contributions of volatilization, thermal degradation, and matrix desorption remain unresolved (130, 131).

Vacuum roasting of Arabica coffee has been investigated at 200 °C and 0.15 kPa (13). At a comparable medium roast degree, the AA content of vacuum-roasted samples was approximately 50% lower than that obtained under atmospheric-pressure roasting, while color and sensory quality remained similar. In coffee, AA forms rapidly during the early stage of roasting and is partly degraded as heating continues. Vacuum conditions therefore alter the dynamic balance among AA formation, removal, and thermal degradation, rather than merely reducing the overall thermal load. Vacuum frying can likewise shorten the high-temperature exposure of potato, fruit, and vegetable chips, although the final mitigation effect depends on system pressure, oil temperature, slice thickness, and final moisture content.

Studies of potato products further show that the effect of vacuum processing depends on where it is introduced into the process. Vacuum frying at 125 °C reduced AA formation in potato crisps by 98% relative to atmospheric frying at 165 °C; however, most panelists preferred the flavor of conventionally fried crisps (132). In reformulated potato chips processed to comparable final moisture contents, vacuum baking reduced AA by 72–98% relative to conventional baking, and the vacuum-baked samples received higher scores for texture and overall acceptability (133). Vacuum treatment may also be applied after heating, although its effectiveness depends strongly on product hydration. At 6.67 Pa and 60 °C, reductions of 43% in biscuits and 18% in potato chips were achieved only after the products had been prehydrated to (a_w > 0.83) (134). These contrasting results indicate that the practical value of vacuum processing cannot be assessed solely by the magnitude of AA reduction; its effects on product flavor and texture, as well as the influence of moisture status on AA removal after heating, should also be considered (135).

#### 5.4.2 Microwave, radio-frequency, infrared, and ohmic heating

Figure 4A highlights the potential of microwave, radio-frequency, and infrared heating to shorten the final drying stage, while also indicating the associated risk of localized hot spots. Microwave, radio-frequency (RF), infrared (IR), and ohmic heating generate distinct temperature and moisture distributions within foods because they deposit energy through different mechanisms (136, 137). Microwave and RF heating produce volumetric heat in materials containing polar molecules through dielectric loss, thereby accelerating internal heating and moisture migration (14, 138, 139). Infrared radiation deposits most of its energy near the material surface, whereas ohmic heating generates Joule heat through the electrical resistance of the food itself (140, 141).

Faster heating alone does not necessarily reduce AA formation. Although electromagnetic and electrothermal technologies can shorten processing times compared with conventional hot-air heating or frying, localized hot spots, edge overheating, and spatial heterogeneity in electrical properties may intensify AA formation in specific regions (142, 143). Therefore, the effectiveness of these technologies depends on whether they can regulate the thermal history and moisture changes actually experienced by the food so as to shorten its residence time within the high-temperature, low-moisture reaction window, without increasing localized thermal loads.

When the same target texture was achieved, microwave heating at 440 W for 3 min reduced AA content by 30.87% compared with conventional baking at 190 °C for 7 min (14). The concentrations of advanced Maillard reaction intermediates and potentially harmful derivatives, including methylglyoxal (MGO), 3-deoxyglucosone (3-DG), and 5-hydroxymethylfurfural (HMF), were also significantly reduced. Increasing the microwave power to 520 W or extending the heating time, however, promoted the formation of AA and other heat-induced by-products. The observed reduction in AA therefore depended on a controlled shortening of thermal exposure (14, 144).

Applying radio-frequency heating as a post-drying step after conventional baking reduced the AA content of cookies from 107.3 to 51.1 ng/g (145, 146). Residual moisture within the product allowed final drying to be completed without prolonged exposure to the high-temperature, low-moisture conditions of conventional baking. Infrared and ohmic heating likewise provide rapid and controllable temperature increases, although infrared radiation has a limited penetration depth, whereas the effectiveness of ohmic heating depends strongly on system conductivity and electrode contact (147–151). Evidence for infrared (IR) heating is more variable, whereas direct studies of ohmic heating remain scarce. During the infrared stabilization of corn grits, microbial contamination was reduced by approximately 5 log cycles, but AA formation increased by 75.5%, indicating that effective microbial decontamination does not necessarily coincide with lower AA levels (152). In carrot-based purées, AA concentrations remained below the method limit of quantification (LOQ, 30 μg/kg) after both ohmic and conventional retort sterilization. Because neither treatment produced quantifiable AA levels, the study could not determine whether ohmic heating reduced AA relative to conventional retorting (153). Across these four heating technologies, AA mitigation is governed primarily by temperature uniformity and the actual thermal history and moisture trajectory experienced by the food, rather than by the heating mode itself.

The studies summarized in Table 3 further indicate that the AA-mitigation effects of the technologies discussed in Sections 5.1–5.4 should be interpreted in relation to the food matrix, treatment sequence, and control conditions. Reported reductions in AA should not be used directly to compare the mitigation efficacy of individual technologies.

**Table 3:** Representative applications and reported acrylamide reductions of emerging and alternative processing strategies in foods.

Processing strategy	Food matrix and intervention stage	Representative treatment conditions	Comparator	Reported AA reduction	Interpretation and evidence boundary	Reference
Pulsed electric fields (PEF)	Lamoka potato chips; pre-frying	1.0 kV/cm; 0.75–1.5 kJ/kg	Matched untreated samples	AA, − 28.9%; frying time, − 10%; oil, − 8%	Precursor removal; single cultivar	(10)
	Sweet-potato kettle chips; pre-frying	1.0 kV/cm; 1.5–3.0 kJ/kg	Matched untreated samples	0.668 μg/g → 0.370 μg/g; − 44.6%	Energy- and matrix-dependent	(105)
PEF + blanching	Sweet-potato chips; pre-frying	1.0 kV/cm, 0.2 s; 85 °C, 5 min	Untreated and single-treatment groups	638 μg/kg; − 46.1%	No significant AA gain over single pretreatments	(197)
PEF + vacuum predrying	Potato chips; pre-frying	600 V/cm, 0.1 s; 30 kPa, 50 °C	No PEF or predrying	2,220 μg/kg → 311 μg/kg; − 85.9%	No significant gain over predrying alone	(196)
Cold atmospheric plasma (CAP)	Potato strips; pre-blanching	DBD, 60 kV, 15 min	No CAP	1807.35 μg/kg → 346.86 μg/kg; − 80.8%	Single matrix; by-products unassessed	(12)
Ultrasound	Potato slices; pre-frying	35 kHz; 95.2 W/kg; 42 °C, 30 min	Direct frying and water soaking	1649.7 μg/kg → 279.3 μg/kg; − 83.1%	Soaking contributed to reduction	(11)
Ultrasound + washing	Potato crisps; pilot-scale washing	1,500 W; 100% amplitude; 2 min	Corresponding wash controls	Up to − 67.1%	Effective only with hot washing	(124)
HPP + asparaginase	Potato strips; enzyme infusion	10,000 ASNU/L; 100–400 MPa, 5 min	HPP or enzyme alone	−26% to −47%	Combined treatment required	(127)
Vacuum roasting	Arabica coffee; roasting	200 °C; 0.15 kPa	Matched atmospheric roasting	Approximately −50%	Laboratory-scale, matched roast degree	(13)
Microwave baking	Biscuits; baking	440 W, 3 min	190 °C, 7 min	−30.87%	Condition-dependent response	(14)
RF post-drying	Cookies; final dehydration	205 °C, 8 min + 27.12 MHz RF	205 °C, 11 min	107.3 → 51.1 ng/g; −52.4%	Heating-schedule dependent	(145)

AA, acrylamide; HPP, high-pressure processing; RF, radio frequency; DBD, dielectric-barrier discharge; ASNU, asparaginase units.

### 5.5 Enzymatic and fermentation-based precursor reduction

#### 5.5.1 Asparaginase treatment

Asparaginase hydrolyzes free asparagine into aspartic acid and ammonia, thereby selectively removing the principal nitrogen-containing precursor of AA (154). Under comparable heating conditions, aspartic acid has a much lower propensity to form AA than asparagine, which gives this enzymatic treatment a high degree of specificity (155). The applied enzyme activity alone, however, does not determine the final mitigation effect; reaction time, pH, temperature, food composition, and effective contact between the enzyme and its substrate are also critical. Asparaginases from different microbial sources vary in stability and optimal reaction conditions, while high salt or sugar concentrations and low water activity can suppress their catalytic activity.

Food microstructure often determines whether enzyme molecules can reach their substrate. Asparagine is relatively accessible in doughs, slurries, and hydrated powder systems, but considerably less so within dense plant tissues such as structurally intact potato strips and coffee beans. In these matrices, resistance to enzyme penetration and diffusion, rather than the enzyme dose itself, is often the principal bottleneck limiting hydrolysis (156).

Residual asparagine after treatment generally correlates more closely with the final reduction in AA than the nominal enzyme dose (157). In raw Arabica coffee beans pretreated with steam and subsequently treated with asparaginase at 5,000 ASNU/kg, the asparagine content decreased by 23.8–34.1% before roasting, while AA content in the roasted product declined by 30.4–41.4% (158). By contrast, the concentration of 4-methylimidazole (4-MEI) remained largely unchanged. This response suggests that the enzyme selectively inhibited the asparagine-dependent branch of AA formation without indiscriminately suppressing other Maillard reaction products.

In intact plant tissues, the primary role of physical pretreatments is not to directly degrade AA, but to reduce mass-transfer resistance between exogenous enzymes and intracellular free asparagine. As discussed in Sections 5.1–5.3, PEF can increase cell-membrane permeability, ultrasound can enhance liquid-phase mass transfer at the tissue–liquid interface, and HPP can alter cell-membrane structure and tissue porosity; all of these effects facilitate the penetration of enzyme solutions into tissues and enhance enzyme–substrate contact (97, 116, 125, 159–161). The HPP–asparaginase findings described above provide direct support for this interpretation, because neither pressurization alone nor asparaginase treatment alone produced a significant reduction in AA (127). When these pretreatments are accompanied by washing, soaking, blanching, or other liquid-contact steps, released asparagine and reducing sugars may also be removed; therefore, the observed reduction in AA should not be attributed entirely to improved enzyme accessibility.

#### 5.5.2 Fermentation-based mitigation

Yeasts and lactic acid bacteria can deplete precursor pools by metabolizing carbohydrates and free amino acids (162, 163). During fermentation, these microorganisms may directly consume glucose, fructose, or asparagine. Organic acids produced through microbial metabolism, including lactic and acetic acids, lower the pH and promote amino-group protonation, thereby limiting the formation of Schiff bases, which are key intermediates in AA formation (164). Some lactic acid bacterial strains also exhibit endogenous asparaginase activity (165), while adsorption of AA onto cell-wall peptidoglycan has been proposed as an additional mechanism (166, 167). Because strains differ in substrate preference, acidification capacity, and fermentation kinetics, the contribution of each mechanism to AA reduction is strain-dependent.

A review of 15 studies covering 41 bacterial and yeast strains reported mean reductions in AA content of 38.33% in bread and 63.87% in fried potato products following fermentation (162). The marked variation among studies reflected differences in strain characteristics, fermentation substrates, and processing conditions. During sourdough fermentation, yeasts and lactic acid bacteria jointly influence sugar consumption, acid accumulation, and the gluten network of the dough. In microbial soaking treatments of potato matrices, by contrast, efficacy depends more strongly on direct surface contact and mass transfer through the liquid phase. The final mitigation effect of fermentation therefore arises from the combined action of several biochemical and physical processes rather than from precursor depletion alone.

### 5.6 Conventional precursor, formulation, and thermal control

Raw-material selection and storage management determine the baseline precursor load before processing (168). Choosing potato and cereal cultivars with lower concentrations of reducing sugars and free asparagine reduces the amount of substrate available for AA formation during subsequent heating (169, 170). Avoiding prolonged storage of potatoes at excessively low temperatures can limit cold-induced sweetening and the associated accumulation of glucose and fructose (8, 171). Subsequent soaking, rinsing, and blanching remove leachable sugars and asparagine from surface tissues (46), with efficacy depending on water temperature, treatment time, liquid-to-solid ratio, and sample thickness. More intensive treatment may remove a greater proportion of the precursors, but it can also increase the loss of water-soluble solids and nutrients (47).

Formulation optimization offers another route for controlling AA. Organic acids lower the pH and protonate the amino group of asparagine, reducing its ability to condense with reducing sugars and form Schiff bases (8, 172). Divalent cations such as calcium and magnesium can interact with asparagine or reactive intermediates (173), while other added free amino acids may compete for reactive carbonyl compounds. Reducing the use of ammonium bicarbonate and other ammonium-containing chemical leavening agents is particularly effective in mitigation formulations for biscuits and expanded foods.

Polyphenols, plant extracts, and natural powders derived from agricultural by-products show less predictable effects on AA formation. They may inhibit AA by scavenging reactive carbonyl compounds, interfering with radical reaction pathways, or altering moisture migration. At the same time, they can introduce additional reactive components, including sugars, amino acids, and organic acids, into the formulation (174–177). In a model wheat bread system, selected polyphenols at inclusion levels as low as 0.1% reduced AA by 16.2–95.2%; however, increasing the quercetin level instead increased AA by 9.8% (178). Similarly, adding 5% pomegranate seed powder to bread significantly reduced AA content, whereas inclusion levels of 7.5% or higher resulted in AA contents greater than those in the 5% treatment (7, 179). A recent Maillard reaction model study likewise found that low levels of phenolic additions, particularly under low-moisture conditions, may increase AA, whereas higher addition levels reduced AA in most of the tested phenolic systems; the direction of this response also varied with initial pH and water content (180). These experimental findings indicate that the effects of plant-derived ingredients on AA formation depend on their chemical composition and inclusion level, as well as the reaction environment and moisture conditions of the system. Antioxidant capacity alone is therefore not a reliable basis for selecting ingredients for AA mitigation.

Conventional thermal-process control strategies include lowering the processing temperature, shortening the heating time, regulating final moisture content, and applying multistage temperature profiles (181–186). AA often accumulates rapidly once the product surface reaches a high-temperature, low-moisture state (187, 188); reducing the time spent within this critical reaction window can therefore limit its formation (189). Lowering the temperature alone, however, is not always effective because the longer processing time required may maintain or even increase the cumulative thermal load. Conversely, insufficient heating may fail to produce the desired color and crisp texture or to ensure microbial safety. Interactions among temperature, time, product thickness, loading density, and moisture migration therefore require thermal-processing parameters to be optimized for the specific food matrix and process configuration (190). In a potato-chip processing study, a combination of raw-material selection, precursor-removal pretreatment, and thermal-process control produced products with AA concentrations below the limit of detection of the HPLC method while maintaining acceptable sensory quality (191).

### 5.7 Integrated and matrix-specific mitigation strategies

#### 5.7.1 Complementary mechanisms in combined treatments

Combined treatments offer the greatest potential for complementary effects when each unit operation addresses a different limiting factor, such as precursor accessibility, solute removal, or thermal exposure (192–195). Table 4 further maps these limiting factors onto representative matrix-specific intervention pathways and specifies the sequence of operations, mechanistic rationale, and evidence boundaries for each pathway. Pulsed electric fields can release intracellular sugars and asparagine, but these compounds remain available for reaction during subsequent heating unless they are removed by rinsing, soaking, or blanching (196). Combining PEF treatment at 1 kV/cm for 0.2 s with blanching at 85 °C for 5 min reduced AA content in fried sweet potato slices by approximately 46.1% (197). Electroporation increased cell-membrane permeability, while blanching provided the aqueous environment and thermal conditions required for precursor extraction. The two treatments are complementary: PEF enhances precursor accessibility, whereas blanching maintains the concentration gradient required for extraction, thereby converting precursor release into net precursor removal. Although PEF-only and blanching-only groups were included, AA levels did not differ significantly among the three pretreatments. Thus, the reported 46.1% reduction supports the tested combined protocol but does not demonstrate an additional benefit or synergy. Mechanistic complementarity alone cannot establish synergy; this would require evidence of an interaction beyond the separate effects under matched final-product endpoints.

**Table 4:** Matrix-specific intervention pathways for acrylamide mitigation and their evidence boundaries.

Food matrix	Primary control target	Representative intervention pathway	Mechanistic rationale	Evidence boundary	Reference
Potato chips	Precursor removal and frying load	PEF → vacuum predrying → frying	Electroporation and reduced final frying load	Laboratory evidence; effects depend on PEF, predrying, and frying conditions	(196)
Potato crisps	Precursor leaching	Ultrasound-assisted cold washing → hot washing → frying	Cavitation-enhanced mass transfer	Pilot-scale effectiveness was inconsistent	(124)
Potato strips	Surface precursors	CAP pretreatment → blanching → frying	Surface modification and reduced precursor availability	Evidence is limited to one food matrix and treatment system	(12)
Food matrix	Primary control target	Representative intervention pathway	Mechanistic rationale	Evidence boundary	Reference
Cookies	Terminal heating load	Recipe modification + partial baking → RF post-baking	Reduced thermal load with color and flavour compensation	Combined intervention; the independent effect of RF was not isolated	(145)
Coffee beans	Roasting environment	Vacuum roasting	Reduced-pressure control of AA formation and removal	Comparisons require a matched roast degree	(13)
Potato strips	Enzyme accessibility	Asparaginase immersion + HPP → frying	Pressure-assisted enzyme infusion	The combined treatment was effective, whereas the individual treatments were not	(127)

AA, acrylamide; PEF, pulsed electric field; CAP, cold atmospheric plasma; HPP, high-pressure processing; RF, radio frequency.

#### 5.7.2 Matrix-specific technology combinations

Figure 4B illustrates a matrix-specific intervention sequence, in which both the choice and order of operations are determined by the dominant limiting factors in the target food system. Combining PEF with vacuum pre-drying can simultaneously alter tissue permeability, initial moisture content, and the subsequent frying time (194, 198). In one combined process, PEF-treated potato slices were vacuum pre-dried before frying at 150 °C, reducing the AA content of the fried product from 2,220 μg/kg to 311 μg/kg, equivalent to a decrease of approximately 85.9% (196). Electroporation enhanced moisture and solute transport, while pre-drying reduced the amount of water that had to be removed during frying, allowing the target texture to be reached in less time. The marked reduction in AA should therefore be attributed to the complete PEF–vacuum pre-drying–frying sequence rather than to PEF treatment alone. However, at the same pre-drying level, adding PEF did not significantly reduce AA relative to pre-drying alone; the 85.9% reduction versus untreated chips therefore should not be interpreted as evidence of synergy.

The combination of ultrasound-assisted soaking and hot-water washing follows the same principle (121, 124). Acoustic cavitation accelerates mass transfer through the liquid phase, but the additional contribution of ultrasound diminishes substantially once hot-water washing has removed most of the accessible precursors. As discussed in Section 5.3.1, this additional effect was not consistently observed when raw-material or washing conditions changed. Ultrasound provides meaningful process intensification only when a sufficient concentration gradient is maintained and the released solutes are promptly removed from the system.

Restricted enzyme penetration in dense plant tissues provides a clear rationale for combining high-pressure processing (HPP) with asparaginase (127, 199). Pressure-induced changes in cell-membrane permeability and tissue porosity can make asparagine located deeper within the matrix accessible to an exogenous enzyme solution. PEF or ultrasound may likewise be combined with asparaginase, although each physical pretreatment modifies the microstructure in a different manner. Technology selection should therefore be guided by the spatial distribution of the substrate and the principal microscale mass-transfer barrier limiting enzyme access.

Emerging alternative heating technologies are often most effective when used as finishing steps rather than as complete replacements for conventional heating. Conventional baking can first establish the basic product structure and characteristic flavor, after which radio-frequency or microwave energy rapidly removes residual moisture from the interior. This sequence prevents low-moisture products from undergoing prolonged high-temperature baking. The radio-frequency post-drying process discussed in Section 5.4.2 illustrates this functionally staged design (145, 146, 200). Biochemical combinations can follow the same principle: asparaginase selectively hydrolyzes asparagine, microbial fermentation consumes reducing sugars, and fermentation-derived acids reduce the reactivity of key intermediates.

Overall, the dominant limiting factors within a food matrix should serve as the primary basis for technology selection. Accordingly, Table 5 compares the principal mitigation strategies discussed in this section in terms of their mechanisms of action and intervention targets, matrix applicability, roles in combined processes, and key limitations. This comparison further supports the design principle illustrated in Figure 4C: combined mitigation is more likely to produce complementary effects only when its individual operations address different limiting factors. When multiple interventions repeatedly act on the same limited precursor pool, additive effects are generally difficult to achieve.

**Table 5:** Mechanism-guided selection of acrylamide mitigation technologies across food matrices.

Technology	Primary mechanism and intervention target	Matrix suitability and processing context	Role in combined processes	Key limitations or selection criteria
Pulsed electric fields (PEF)	Electroporation → promotes the release of intracellular precursors	Intact potato and sweet potato tissues; pre-frying treatment	Applied before washing or blanching; can be coupled with vacuum predrying	Requires liquid-phase removal of precursors; affected by matrix properties and energy input
Ultrasound	Cavitation → enhances the leaching of water-soluble precursors	Sliced products subjected to soaking, washing, or blanching	Enhances mass transfer during aqueous extraction	Requires maintenance of a concentration gradient; affected by equipment configuration and washing conditions
High-pressure processing (HPP) combined with asparaginase	Increased tissue permeability → improved enzyme–substrate contact	Dense plant tissues in which enzyme penetration is constrained	Pressure-assisted enzyme infiltration before heating	May soften tissues; pressure and enzymatic treatment conditions are critical
Cold atmospheric plasma (CAP)	Surface reactions involving reactive oxygen and nitrogen species (RONS) → modification of precursors and microstructure	Foods with readily accessible surfaces; pretreatment before blanching or frying	Surface pretreatment	Effects are mainly limited to the surface; few studies have directly measured AA in food matrices; by-products require evaluation
Plasma-activated water (PAW)	Liquid-phase reactive species → alteration of Maillard reaction–related processes	Foods amenable to soaking, washing, or spraying	Candidate aqueous pretreatment	AA was not directly quantified in the cited food study; reactive composition depends on preparation and storage conditions
Vacuum frying, baking, or roasting	Reduced water boiling point → altered moisture removal and thermal history	Crisp snacks, baked or roasted products, and coffee	Primary heating approach or moisture-dependent post-treatment	Dependent on processing stage, pressure, temperature, and final moisture content; possible quality trade-offs
Microwave, radio-frequency (RF), infrared (IR), and ohmic heating	Energy deposition → regulation of final-stage drying and localized thermal exposure	Baked foods and systems requiring directed heating	Targeted heating or final-stage treatment	Localized hot spots and nonuniform heating may occur; performance depends on matrix-specific energy absorption and heat transfer; evidence remains limited for some modalities
Asparaginase and fermentation	Asparagine hydrolysis, sugar consumption, and acidification → reduced precursor load	Dough and batter systems and plant tissues accessible to enzymes	Treatment before final heating; combined with physical pretreatments where necessary	Depends on substrate accessibility, reaction conditions, fermentation performance, and sensory quality
Raw-material selection, formulation, and conventional thermal-process control	Control of precursor load and the thermal reaction window	Potatoes, cereals, baked foods, and other thermally processed foods	Foundational control before applying targeted emerging technologies	Must balance color, flavor, texture, nutritional quality, and microbial safety

AA, acrylamide; CAP, cold atmospheric plasma; HPP, high-pressure processing; IR, infrared; PAW, plasma-activated water; PEF, pulsed electric field; RF, radio frequency; RONS, reactive oxygen and nitrogen species.

### 5.8 Regulatory context and industrial feasibility

Commission Regulation (EU) 2017/2158 requires relevant food business operators to implement AA mitigation measures and monitor their effectiveness, with the aim of keeping AA concentrations as low as reasonably achievable and below the benchmark levels for the relevant food categories (201). These benchmark levels include 500 μg/kg for ready-to-eat French fries, 750 μg/kg for potato chips, 350 μg/kg for biscuits and wafer products, and 400 μg/kg for roasted coffee. These values are benchmarks for evaluating mitigation performance, rather than statutory maximum limits (201). Codex CXC 67–2009 provides relevant guidance on raw materials, ingredients, and heating processes, while emphasizing that AA reduction should not compromise microbial safety, nutritional quality, or consumer acceptability (202).

Table 5 summarizes the mechanisms of action of the various technologies; for industrial application, it is also necessary to consider where each treatment can be introduced into existing production lines. In potato processing, PEF can be applied before washing or blanching, ultrasound can be integrated into aqueous processing steps, and HPP-assisted asparaginase treatment requires the addition of enzyme-contact and pressure-cycle steps before frying. RF post-drying has been evaluated following conventional baking in some applications (145, 146). In contrast, when vacuum frying or vacuum baking is used as the primary heating step, modifications to core heating equipment are required.

One experimental study on French fries reported lower AA concentrations in fried products after CAP pretreatment for 5–15 min (12); however, whether this treatment can achieve both adequate throughput and uniformity on industrial production lines remains to be validated. The cited PAW food study did not measure AA, and PAW therefore cannot yet be regarded as an established AA-control operation on this basis (112). Validation under production-line conditions should consequently assess production efficiency and energy consumption and, where applicable, water use, soluble-solids loss, oil uptake in fried products, and sensory quality, as well as potential by-products arising from reactive treatments. AA-mitigation effects should be compared at matched final-product endpoints.

## 6 Conclusions and future research priorities

### 6.1 Conclusion

The mitigation of AA in thermally processed foods depends not only on the technology applied, but also on food composition, matrix structure, and the thermal history and moisture changes experienced during processing. The same treatment may therefore yield different outcomes in different food matrices or when applied at different stages of the same process.

Rather than viewing AA mitigation as the search for a universally optimal technology, it should be considered a process-design problem constrained by multiple conditions. A suitable intervention should address the dominant limiting factors in the target product while maintaining the required moisture state, color, texture, flavor, and food-safety outcomes. Because reported reductions in AA are influenced by the food matrix, precursor load, intervention stage, complete processing sequence, control conditions, and whether final product endpoints are matched, reported percentage reductions are not inherent measures of technological performance and should not be compared in isolation from these conditions. Moreover, a reduction in AA at the product level does not necessarily translate into a proportional reduction in dietary exposure or establish the magnitude of any health benefit; these outcomes also depend on food-consumption patterns and the contribution of the food to total dietary AA intake.

Combined treatments are most clearly justified when multiple interconnected limiting factors are present in the target product and cannot be addressed simultaneously by a single operation while maintaining the intended product endpoints. Their value lies not in increasing the number of interventions, but in addressing distinct bottlenecks in AA formation through an appropriately ordered sequence of operations. Repeated interventions directed at the same limited precursor pool are unlikely to yield substantial additional benefits. In contrast, appropriately sequenced interventions are more likely to achieve consistent mitigation when their individual operations address different remaining limiting factors. In practical processing, the more meaningful comparison is not which technology produces the greatest individual reduction, but which process sequence controls AA formation while maintaining the intended product endpoints. Selecting strategies according to this principle can reconcile AA mitigation with the sensory attributes and food-safety functions that thermal processing must preserve.

### 6.2 Future research priorities

Future studies should quantitatively distinguish the contributions of individual unit operations within combined treatments to the final reduction in AA, rather than inferring those contributions from the overall treatment outcome alone. For PEF-assisted frying, a key question is how much of the final reduction arises from electroporation-enhanced precursor release and removal, and how much arises from shorter frying times, altered dehydration, or changes in oil uptake. For CAP, it remains necessary to determine which reactive species can reach and persist at food surfaces, and whether their effective doses can be controlled to achieve reproducible precursor modification and AA reduction across raw-material batches. For dense plant tissues, the minimum combination of tissue permeability, enzyme concentration, and contact time needed to achieve effective asparagine hydrolysis should be established. The need for such mechanistic attribution and reproducibility validation applies equally to other mitigation technologies. Their direct effects, key determinants, and relationships with subsequent operations should be defined within the relevant food matrices and processing sequences.

Quantitative interpretation of the contributions of individual unit operations must also be validated across food matrices and processing contexts. Matrix-specific validation should not be restricted to a single cultivar, formulation, or laboratory-scale process. For alternative heating technologies, studies should determine the thermal histories and moisture-change trajectories that reduce time spent under AA-forming conditions while avoiding local overheating or impairment of required product attributes. Accordingly, comparisons among mitigation strategies should be made at matched endpoints for final moisture content, color, texture, and, where relevant, microbial safety. Such comparisons are necessary to distinguish genuine AA mitigation from apparent reductions caused by under processing or differences in final product state.

Future evaluations of how AA reduction improves dietary exposure should integrate directly measured AA concentrations in finished products with actual food-consumption data. Where feasible, hemoglobin adducts and urinary metabolites may supplement dietary exposure estimates, but the effects of smoking, occupational exposure, and metabolic variability must be considered. Uncertainty in food AA concentrations and intake estimates should also be explicitly reported to indicate the extent to which AA reduction during processing may translate into lower dietary exposure.

Methodological rigor is the shared prerequisite for the mechanistic attribution, cross-matrix comparison, and exposure interpretation described above. Studies should characterize the actual treatment intensity applied to foods and its process context, including the initial precursor state, key subsequent operations, and the actual thermal history and moisture changes, rather than inferring these from nominal equipment parameters or processing time alone. Conclusions on AA mitigation should be supported by validated quantitative measurements suitable for the relevant food matrix and tested for robustness under raw-material and process variations that approximate real production. Such evidence is needed to define the effective operating range and failure conditions and to determine whether apparent additional effects in combined treatments result from complementary operations, rather than differences in processing intensity or final product state.
