# Supplementary material for: Mechanism-guided mitigation of acrylamide in thermally processed foods: emerging technologies and analytical verification
Source: Front Nutr. 2026 Oct 20;13:1981497. doi: 10.3389/fnut.2026.1981497 (PMC13729798; doi:10.3389/fnut.2026.1981497)
Supplement: Supplementary file 1 [file Supplementary_file_1.DOCX]

Supplementary Material

# 1 Supplementary Methods

## 1.1 Literature Search

The literature underpinning this narrative review was identified through multiple iterative search rounds. The final search was completed in August 2026, with no restriction on publication year. An initial broad search was conducted in Web of Science and PubMed using terms related to acrylamide, thermally processed foods, processing-related hazards, pretreatment technologies, analytical methods, and mitigation measures. Titles and abstracts were then assessed for relevance to acrylamide toxicity and metabolism, formation processes, analytical verification, mitigation technologies, and related mechanisms. Duplicate records and publications outside the scope of this review were excluded. More targeted searches were subsequently conducted in Google Scholar by combining acrylamide-related keywords with the names of specific processing technologies, including pulsed electric field and cold atmospheric plasma. Reference lists of relevant publications were also consulted, and further targeted searches were conducted to identify studies supporting the discussion of specific mechanisms and processing operations.
